# Design and Evaluation of a Mobile App for Intergenerational Communication: User-Centered Participatory Design and Experimental Mixed Methods Study

**DOI:** 10.2196/75950

**Published:** 2025-09-17

**Authors:** Soondool Chung, Hannah Lee, Jeehye Jung

**Affiliations:** 1 Department of Social Welfare Ewha Womans University Seoul Republic of Korea; 2 Ewha Institute of Intergeneration for Age Integration Research Ewha Womans University Seoul Republic of Korea

**Keywords:** intergenerational communication, digital inclusion, user-centered design, mobile app development, technology acceptance model, intergenerational programs, social connectedness

## Abstract

**Background:**

Social isolation and weakened intergenerational ties pose significant threats to the emotional well-being and social support networks of older adults. Although structured intergenerational programs can reduce age-related stereotypes and promote connectedness, their accessibility is often hindered by physical and logistical constraints. The increasing digital literacy among older populations presents new opportunities for technology-based interventions to support meaningful cross-generational engagement.

**Objective:**

This study aimed to design and evaluate a mobile app that fosters intergenerational communication and enhances perceived social support in older adults using a user-centered design framework grounded in the double diamond model.

**Methods:**

The development process followed the 4 phases of the double diamond model. In the discover phase, surveys with older and younger adults identified distinct usability preferences. The define phase synthesized these insights into key design principles. In the develop phase, a prototype was created and iteratively refined through usability testing. Finally, in the deliver phase, a 2-week experimental study involving 39 participants (20 older adults aged 68-82 years and 19 younger adults aged 22-39 years) assessed changes in intergenerational interaction, perceived social support, and user satisfaction.

**Results:**

The app appeared to enhance intergenerational communication and perceived social support, particularly among older participants. Users reported increased comfort and emotional connection in cross-generational conversations. Accessibility features and engaging content were noted as contributing to positive user experiences across age groups.

**Conclusions:**

This study suggests the potential of user-centered digital platforms to promote social well-being among older adults. By addressing the unique needs of multiple generations, such interventions may help foster inclusive digital environments and contribute to age-friendly, connected societies. Despite limitations related to sample size, duration, and cultural context, the study provides preliminary evidence for the potential of co-designed digital tools in supporting intergenerational communication and aging-in-place.

## Introduction

Intergenerational conflict has emerged as a critical social issue, undermining societal cohesion beyond mere differences in opinions between older and younger generations. Such conflicts manifest as misunderstandings, stereotypes, discriminatory attitudes, and competition for resources, resulting in tensions that hinder effective intergenerational collaboration and societal harmony [1,2]. This phenomenon is prevalent across various cultural contexts and is often exacerbated by the increasing generational divide, which limits opportunities for mutual understanding and interaction [2,3]. According to social identity theory [4], individuals tend to categorize themselves into in-groups and out-groups, fostering biases and discriminatory attitudes toward those outside their group. The generational divide intensifies such perceptions, leading to heightened tensions and societal fragmentation.

In preindustrial societies, intergenerational communication was inherently facilitated by extended family structures and community-based living arrangements, where multiple generations coexisted and engaged in shared responsibilities. However, the shift toward nuclear family living arrangements and the advent of digital media have significantly reduced these opportunities, reinforcing psychological and physical distances between age groups. This decline in direct contact between age groups has contributed to social alienation and hindered the formation of intergenerational trust. According to the contact hypothesis [5], sustained intergroup contact reduces prejudice and promotes positive relationships. This has been empirically demonstrated in structured intergenerational programs, which consistently reduce stereotypes and foster mutual respect [6,7].

To address the widening generational divide, intergenerational programs have been widely implemented to facilitate direct engagement between older and younger generations. Such programs yield mutual benefits; older adults experience enhanced emotional well-being, cognitive stimulation, and increased social participation, while younger participants benefit from identity formation and skill acquisition [8]. Despite their well-documented advantages [9], face-to-face engagement remains constrained by physical distance, mobility limitations, and logistical barriers [10,11].

Digital communication tools offer a scalable and accessible alternative, particularly for populations with restricted opportunities for in-person interaction. The COVID-19 pandemic further accelerated digital adoption among older adults; in Germany, for instance, internet use among older individuals increased from 58% to 81% during the pandemic [12]. Moreover, empirical evidence suggests that even indirect or extended intergenerational contact can reduce age-based prejudice among younger people [7], underscoring the potential of digital platforms to foster intergenerational understanding. These findings highlight the value of digital environments as a means of promoting meaningful engagement across generations, extending the reach and impact of conventional face-to-face programs.

In line with this shift, various digital interventions have emerged globally to enhance intergenerational engagement. For example, Müller et al [13] developed a web-based platform to improve communication between older adults and their families through shared modules and collaborative learning. Similarly, Phang et al [14] identified diverse programs using accessible technologies—such as smartphones, video calls, and messaging apps—to strengthen emotional connection and intergenerational bonding. However, most existing platforms lack mechanisms for sustained, bidirectional interaction. Although older adults’ digital literacy has increased [14], many platforms still fall short of addressing the nuanced needs and co-design preferences of both age groups. This gap underscores the need for inclusive, user-centered digital solutions that enable emotionally reciprocal and goal-oriented communication across generations.

In response to these limitations, this study introduces CoGen (Ewha Institute for Age Integration Research), a mobile app designed to promote intergenerational communication. Unlike prior platforms that rely on structured prompts or 1-way content delivery, CoGen supports user-generated, bidirectional interaction designed to accommodate the nuanced needs and co-design preferences of both younger and older users. To achieve this, CoGen’s development is anchored in user-centered design (UCD) principles [15,16] and operationalized through the double diamond model [17], a 4-phase process (discover, define, develop, and deliver) that facilitates a structured, iterative approach for identifying user needs and refining design solutions in response to ongoing feedback. Implicit in this design philosophy is the contact hypothesis [5], which posits that sustained interaction under conditions, such as equal status, common goals, intergroup cooperation, and institutional support, can reduce prejudice. While this study does not directly test the hypothesis, its framework serves as a useful theoretical lens for understanding how intergenerational communication—especially in structured digital settings—might contribute to prejudice reduction.

Rather than simply developing a communication tool, this study aims to create CoGen as a user-centered, age-inclusive digital environment that enables users to exchange experiences, share cultural knowledge, and engage in collaborative activities. By fostering sustained and meaningful digital interaction, the project seeks to bridge the intergenerational divide, enhance older adults’ social connectedness, and support global efforts toward digitally inclusive aging. In doing so, the development and evaluation of CoGen align with international movements advocating for scalable, human-centered technologies that empower older populations and promote equitable participation.

## Methods

### Double Diamond Model and Research Process

**Figure 1:**
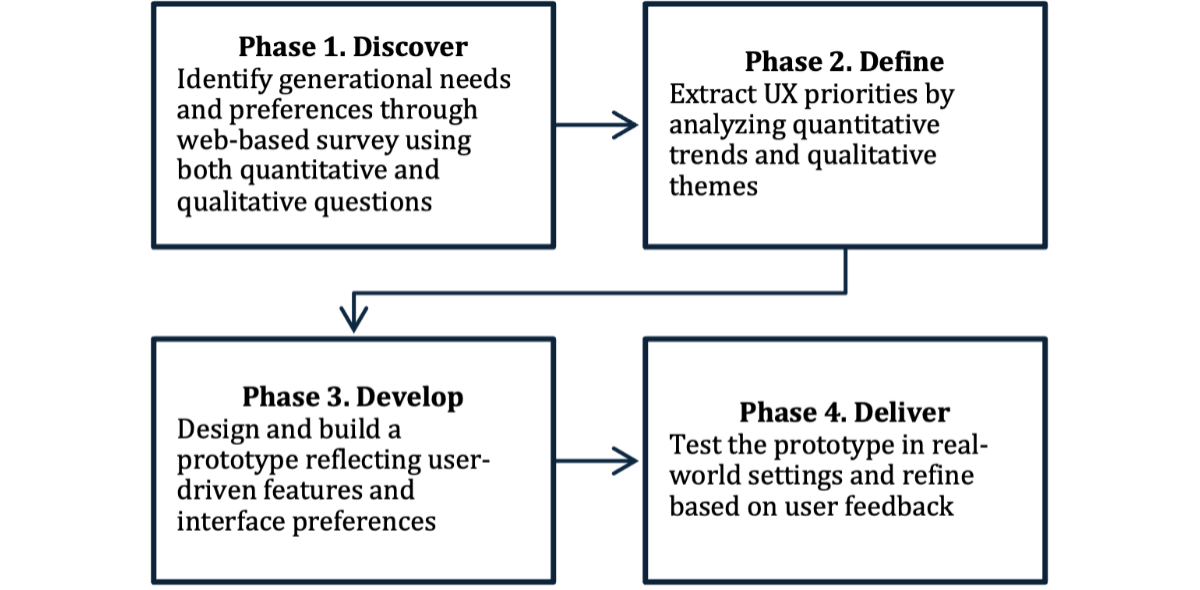
User-centered app development for intergenerational communication using the double diamond model. UX: user experience.

To guide the design and evaluation of the proposed mobile app, this study adopts the double diamond model [17], a 4-phase framework comprising discover, define, develop, and deliver. Alternating between divergent and convergent thinking, this model enabled a user-centered, iterative approach to address challenges in intergenerational digital communication. Each phase incorporated continuous input from both younger and older users to ensure that the final design was both accessible and responsive to real-world needs. Figure 1 presents a visual summary of the study design, showing the sequential flow of the 4 phases along with key methods and objectives associated with each phase.

### Phase 1: Discover: User Research

#### Research Focus

In the discover phase, the technology acceptance model [18] guided the identification of key factors influencing user attitudes and adoption intentions across age groups. A cohort-specific survey approach was used to capture generational differences in communication needs, motivations, and feature preferences. By incorporating both usability concerns of older adults [19] and engagement drivers for younger users [20], this phase laid the groundwork for a user-centered app design.

#### Participants and Research Procedure

Recognizing the distinct usability barriers faced by older adults and the engagement drivers influencing younger adults, this study recruited 2 age groups: younger adults (20-39 years) and older adults (60-79 years). A nationwide web-based survey was conducted in South Korea, using proportional stratified random sampling from an existing panel database managed by a professional research firm. Stratification criteria included age, gender, and prior digital communication experience to ensure demographic diversity within each group. Email invitations outlining the study’s purpose were sent, and a total of 413 participants—202 younger adults and 211 older adults—voluntarily completed the survey.

#### Measures and Data Collection

##### Measurement Approach

To assess participants’ attitudes, expectations, and key adoption factors for an intergenerational communication app, we used a mixed methods approach with quantitative survey items and qualitative open-ended responses. Our measurement strategy primarily used single-item indicators, carefully adapted from established multi-item scales for their high face validity and relevance [21-27]. This approach prioritized feature-level granularity over composite scoring, aligning with our exploratory UCD goals. Furthermore, we followed prior methodological support for single-item measures when constructs are narrow, concrete, and easily understood [28-30]. As these items were not intended to form reflective scales, internal consistency metrics like Cronbach α were not computed.

##### Attitudes Toward Intergenerational Communication

Participants’ overall attitudes toward intergenerational communication were assessed using an adapted version of the scale by Lee and Lee [21]. Two conceptually distinct items measured interest and perceived importance (eg, “How interested are you in communicating with other generations?” and “How important do you think it is to have communication between generations?”), rated on a 5-point Likert scale (1=not at all and 5=very much).

##### Perceived Necessity and Willingness to Use an Intergenerational Communication App

To assess both the perceived necessity of an intergenerational communication app and participants’ willingness to use it, two key items were included: (1) “How necessary do you think an app for intergenerational communication is?” (1=not necessary at all and 5=very necessary). (2) “If an app for intergenerational communication were developed, how often do you think you would use it?” (1=not at all and 5=very often). These items aimed to evaluate both the demand for such an app and participants’ likelihood of adoption.

##### Motivational Factors for App Use

This section examined the anticipated use cases of the app, including social communication (eg, maintaining relationships across generations), entertainment (eg, engaging in shared recreational activities), information exchange (eg, learning about generational perspectives), and emotional support (eg, alleviating social isolation). Participants rated 8 motivational factors on a 5-point Likert scale (1=not relevant at all and 5=very relevant) to assess their perceived significance. These factors included social communication, passing time (relieving boredom), entertainment and enjoyment, exchanging necessary information or opinions, reducing loneliness, health management, discussing concerns not easily shared with same-generation peers, and cultural exchange. The items were adapted from Lee and Na [22] and Jun et al [23] to align with prior research on intergenerational communication and digital engagement.

##### Preferred App Features

Guided by Davis’s [18] technology acceptance model, participants evaluated both content and design attributes likely to influence perceived ease of use and perceived usefulness of the app. For perceived ease of use, 7 items were assessed (eg, ease of access to content, clarity and accuracy of information, and simplicity of design). For perceived usefulness, 9 items were included (eg, convenience, sense of belonging, emotional satisfaction, and health relevance). These items were adapted from prior studies on digital content evaluation and intergenerational engagement [24,25].

##### Aesthetic and Gamification Components

Six aesthetic and gamification components were assessed to evaluate their perceived importance in enhancing user engagement. These included rewards (eg, points, badges, and levels), customization options (eg, avatars and profiles), friend integration (eg, KakaoTalk and Facebook), immersive meeting spaces (eg, augmented reality or virtual reality), visual appeal (eg, icons and backgrounds), and auditory appeal (eg, sounds and music). All items were rated on a 5-point importance scale (1=not important at all and 5=very important). The items were adapted from Choi et al [26] and Kim and Kim [27] to ensure consistency with prior research on digital engagement and user experience (UX) design.

##### Qualitative Responses on Cross-Generational Information and Conversation Topics

In addition to structured survey items, participants provided open-ended responses about specific topics and types of information they believed would best facilitate meaningful intergenerational interactions. These qualitative insights supplemented the quantitative findings, ensuring that the app’s design aligns with real-world conversational preferences.

#### Statistical and Thematic Analysis

Quantitative data were analyzed using descriptive statistics and independent-samples 2-tailed *t* tests to examine generational differences across key variables, including perceived necessity, willingness to use the app, motivational factors, and preferences for content and design features. Mean comparisons allowed for the identification of statistically significant age-related patterns in user needs and expectations.

In parallel, qualitative data from open-ended survey responses were analyzed using inductive thematic analysis. One researcher (JJ) conducted an initial review of all responses to identify semantically similar ideas and organized them into preliminary categories. These were grouped into broader conceptual themes through a data-driven approach—for example, responses referencing real estate, stock investments, and financial planning were grouped under “finance,” while those related to exercise, dieting, and eating habits were categorized under “health.” To enhance the credibility of the analysis, a second researcher (HL) independently reviewed the categorized data and cross-checked the thematic structure. Discrepancies in categorization were discussed and resolved collaboratively.

### Phase 2: Define: UX Design Extraction Through Data Analytics

In the define phase, findings from the quantitative and qualitative analyses conducted in the discover phase were synthesized to establish key UX objectives and design requirements. This phase focused on translating user insights into actionable design principles, aligning core functionalities and content strategies with generational communication patterns and technological expectations. No new analyses were conducted; instead, this phase served as a bridge between empirical findings and UCD decisions.

### Phase 3: Develop: Prototype Development and UX Optimization

#### Design Objectives

This phase focused on translating key insights from the define phase into a functional prototype that supports intergenerational communication through an intuitive, engaging, and accessible design. The primary goal was to develop an interface that balances structured discussions with active engagement while ensuring seamless navigation for both younger and older users. The prototype underwent preliminary usability testing to validate design decisions and inform further refinement.

#### User Interface or UX Design and Wireframing Strategy

In the develop phase, findings from the define stage were translated into concrete interface components through low- and high-fidelity prototyping. Design decisions were guided by age-specific user preferences identified in previous phases. For older adults, design emphasis was placed on simplicity, clarity, and visual accessibility, including large font sizes and high-contrast modes. For younger adults, vibrant color palettes and dynamic layouts were incorporated to enhance visual engagement and usability.

Wireframing began with a streamlined home screen comprising 5 core functions to reduce cognitive load while maintaining structural clarity. To support long-term engagement, visual elements such as level indicators and progress bars were introduced, emphasizing intuitive feedback mechanisms.

#### Prototype Feature Integration Plan

Based on the motivational and usability patterns revealed in the discover and define phases, a set of core features was developed. These included theme-based discussion boards, digital conversation prompts, proprietary 1:1 friend matching, and progression-based gamification. Design mapping was used to align each feature with specific user needs—for example, older adults’ emphasis on meaningful social bonding informed the inclusion of noncompetitive reward systems, while younger adults’ preference for entertainment-driven experiences led to the development of dynamic prompts and visual engagement indicators.

#### Preliminary Usability Testing Procedure

Before broader evaluation, a pilot usability test was conducted to evaluate the prototype’s interface, features, and overall usability. In total, 8 participants—5 younger adults (aged 20-39 years) and 3 older adults (aged 60-79 years)—were recruited to test core functions including onboarding, feature navigation, and emotional appeal. Feedback was collected through structured observation and posttask interviews. Key points of friction and areas of positive engagement were identified to guide iterative improvements before moving into the final testing phase.

### Phase 4: Deliver: Usability Testing and User-Centered Refinement of CoGen

#### Phase Overview

The deliver phase aimed to evaluate the CoGen prototype’s effectiveness in fostering intergenerational engagement in real-world settings. A 2-week experimental study using a mixed methods approach was conducted to assess UX, communication outcomes, and app usability.

#### Study Design and Procedures

A total of 39 participants (17 men and 22 women) aged 20 to 82 (mean 48.57, SD 24.56) years took part in a 2-week experimental study conducted from October 28 to November 12, 2024. The sample included 20 older adults (aged 68-82 years) recruited from 2 senior welfare centers in Seoul and 19 younger adults (aged 22-39 years) recruited via web-based advertisements. To minimize usability barriers, only older adults who owned and regularly used smartphones were eligible to participate.

Prior to the intervention, all participants attended an orientation session held at a welfare center in Seoul, with simultaneous Zoom (Zoom Video Communications) access for those unable to attend in person. The orientation lasted approximately 1 hour and included an explanation of the study purpose and procedures, followed by the administration of the preintervention survey. Over the subsequent 2 weeks, participants were instructed to log in daily to the CoGen app, post or respond to discussion prompts, and engage with others through comments and reactions.

Following the 2-week use period, participants completed a postintervention survey. Subsequently, focus group interviews (FGIs) were conducted to gather qualitative feedback on UX, perceived value, and emotional engagement. All 39 participants were divided into 7 small groups (fewer than 6 participants per group), and each group participated in a single FGI session, resulting in 7 sessions in total. Interviews took place in November 2024, either in person at senior welfare centers or on the web via Zoom, depending on participant accessibility. All younger adults participated through web-based sessions, whereas older adults primarily attended in person unless remote participation was required. Each session lasted approximately 1 hour and was moderated by a lead researcher (HL), with a second researcher (JJ) observing and taking notes. Participants provided both verbal and written consent for audio recording and transcription. A structured open-ended question guide was used to explore experiences of intergenerational communication, app usability, emotional responses, and perceived facilitators and barriers.

#### Measures

##### Postuse App Evaluation and Behavioral Intentions

Participants’ postuse evaluation of the app and their behavioral intentions were assessed using a multidimensional measurement strategy comprising both multi-item and single-item indicators, all rated on 5-point Likert scales. Items were adapted from instruments used in the discover phase and modified to capture users’ emotional, cognitive, and behavioral responses after 2 weeks of app use. Single-item measures were selected for their clarity and conceptual precision, consistent with prior methodological recommendations [28-30].

##### Affective and Overall Evaluation

To evaluate affective aspects of intergenerational communication facilitated through the CoGen app, participants were asked to rate their comfort during cross-generational conversations (“How comfortable did you feel while having conversations with individuals from other generations using the CoGen app?”) and their satisfaction with those conversations (“How satisfied were you with the intergenerational conversations through the app?”). These items captured users’ emotional responses and overall impressions of the app-mediated interaction.

##### Emotional Engagement

Emotional engagement was measured through 4 items: “enjoyment,” “happiness,” “interest,” and “a sense of similarity or affinity with others.” These were presented under the prompt, “To what extent did you experience the emotions or values you had expected from using the intergenerational communication app?” and were designed to evaluate participants’ subjective emotional experiences during app use.

##### Functional Usability

Functional usability was measured using 2 items: “It was easy to find the information I needed in the CoGen app” and “The information and content provided in the app were easy to understand.” These were also rated on a 5-point Likert scale (1=strongly disagree and 5=strongly agree). All items were analyzed individually, and descriptive statistics were used to examine users’ perceptions of the app’s usability, clarity, and interface design.

##### Perceived Necessity, Use Intentions, and Recommendation Willingness

Participants’ attitudes toward the CoGen app were assessed using 3 single-item measures, each rated on a 5-point Likert scale. Two items (eg, “Do you think the CoGen app is necessary?” and “Do you intend to continue using the CoGen app in the future?”) were administered both before and after the intervention to assess perceived necessity and continued use intention, respectively. An additional item (eg, “Would you recommend the CoGen app to others?”) was included in the postintervention survey to assess participants’ willingness to recommend the app to others.

##### Intergenerational Communication

Changes in intergenerational communication were measured using 6 items administered before and after the 2-week intervention, rated on a 5-point Likert scale (1=strongly disagree and 5=strongly agree). Items were adapted from the European Values Study and the revised Solidarity Scale [31,32], modified for app-based cross-generational contexts. Intergenerational interaction was assessed with 2 items (pre: α=.76; post: α=.79), such as “I now talk with members of other generations more frequently than before.” Intergenerational understanding was measured with 4 items (pre: α=.87; post: α=.91), including “I have come to respect the culture of other generations” and “I have come to understand the realities faced by other generations.”

##### Psychosocial Outcomes

Perceived social support was assessed using 8 items from the Medical Outcomes Study Social Support Survey [33], rated on a 5-point Likert scale (1=strongly disagree and 5=strongly agree) before and after the intervention (pre: α=.91; post: α=.93). Example items include “There is someone who makes me feel cared for” and “I have someone to talk to about work or household problems.”

Depressive symptoms were measured using the 10-item short form of the Center for Epidemiologic Studies Depression Scale, validated in Korean [34], with responses rated on a 5-point scale (1=not at all and 5=almost always; pre: α=.83; post: α=.85). Sample items include “I felt depressed” and “I had trouble concentrating on what I was doing.”

#### Data Analysis

A mixed methods approach was used to analyze both quantitative and qualitative data. For the quantitative component, paired-sample 2-tailed *t* tests were conducted to compare participants’ pre- and postintervention responses on key outcome variables, including intergenerational interaction, perceived understanding, social support, and depressive symptoms. Descriptive statistics were also used to summarize postuse evaluations, usability perceptions, and emotional engagement indicators.

For the qualitative component, transcripts were analyzed using thematic analysis [35]. Three members of the research team—including 2 senior researchers with expertise in aging and 1 qualitative methods specialist—independently reviewed transcripts and field notes. An inductive coding process was used to identify recurring patterns and refine overarching themes such as perceived benefits, design-related frustrations, and recommendations for improvement. Triangulation and consensus meetings were used to enhance the trustworthiness and analytical rigor of the findings.

### Ethical Considerations

This study received institutional review board approval from Ewha Womans University (IRB ewha-202203-0022-01). All participants provided written informed consent prior to participation, and data were anonymized to ensure confidentiality. Identifiable information was removed during transcription and analysis to protect participant privacy. As compensation, each participant received a gift voucher worth approximately US $15.

## Results

### Phase 1: Discover; Key Findings From User Research

#### Attitudes Toward Intergenerational Communication

Older adults exhibited significantly higher interest in intergenerational communication (mean 3.87, SD 0.63) compared to younger adults (mean 3.61, SD 0.81; t_411_=–3.63; *P*<.001; *d*=0.36). They also placed greater importance on exchanging necessary information (mean 4.05, SD 0.66) than younger adults (mean 3.86, SD 0.82; t_411_=–2.59; *P*=.01; *d*=0.26).

#### Perceived Necessity and Willingness to Use an Intergenerational Communication App

Older adults rated the perceived necessity of an intergenerational communication app significantly higher (mean 3.82, SD 0.73) than younger adults (mean 3.47, SD 0.83; t_411_=–4.54; *P*<.001; *d*=0.45). They also demonstrated a greater willingness to use the app (mean 3.81, SD 0.71) compared to younger adults (mean 3.17, SD 0.87; t_411_=–8.17; *P*<.001; *d*=0.81).

#### Motivational Factors for App Use

Distinct differences emerged in how and why users expected to use the app. Older adults were more likely to use the app for social communication (mean 3.87, SD 0.63) than younger adults (mean 3.61, SD 0.81; t_411_=–3.63; *P*<.001; *d*=0.36), indicating a stronger interest in maintaining intergenerational relationships. They also placed greater importance on passing time (mean 2.97, SD 0.86) than younger adults (mean 3.19, SD 1.01; t_411_=2.38; *P*=.018; *d*=–0.23), although the difference favored younger adults. In addition, older adults expressed significantly greater interest in entertainment and enjoyment (mean 3.50, SD 0.73) than younger adults (mean 3.19, SD 1.03; t_411_=–3.52; *P*<.001; *d*=0.35).

Regarding exchanging necessary information or opinions, older adults again rated this higher (mean 4.05, SD 0.66) than younger adults (mean 3.86, SD 0.82; t_411_=–2.59; *P*=.01; *d*=0.26). They also viewed the app as more useful for reducing loneliness (mean 3.10, SD 0.89) than younger adults (mean 2.89, SD 1.12; t_411_=–2.10; *P*=.04; *d*=0.21) and placed greater emphasis on health management (mean 3.77, SD 0.88) compared to younger adults (mean 3.31, SD 1.02; t_411_=–4.90; *P*<.001; *d*=0.47).

Younger adults, on the other hand, reported significantly greater interest in discussing concerns not easily shared with same-generation peers (mean 3.59, SD 0.94) than older adults (mean 3.06, SD 0.94; t_411_=5.73; *P*<.001; *d*=–0.56). Finally, older adults rated cultural exchange significantly higher (mean 3.72, SD 0.72) than younger adults (mean 3.44, SD 0.94; t_411_=–3.39; *P*=.001; *d*=0.33), suggesting a stronger openness to learning about different generational perspectives.

#### Preferred App Features

##### Perceived Ease of Use

Both younger and older adults highly prioritized the reliability of provided information (younger adults: mean 4.26, SD 0.86 and older adults: mean 4.41, SD 0.66), with older adults showing a marginally higher preference (t_411_=–1.98; *P*=.048; *d*=0.20). Similarly, clarity and accuracy of information received high, comparable ratings from both groups (younger adults: mean 4.16, SD 0.83 and older adults: mean 4.26, SD 0.68; t_411_=–1.34; *P*=.18; *d*=0.13).

A significant difference emerged in the ease of access to information, where older adults placed notably higher importance (mean 4.14, SD 0.60) than younger adults (mean 4.00, SD 0.73; t_411_=–2.12; *P*=.03; *d*=0.21), underscoring the need for an intuitive and navigable interface for this demographic. While simplicity and readability of content were also more valued by older adults, this difference was not statistically significant.

Both groups rated the relevance, appropriateness, and immediate accessibility of content similarly, with no significant differences. Interestingly, younger adults placed slightly more emphasis on engagement and entertainment value, though this difference was not significant.

##### Perceived Usefulness

Older adults consistently reported significantly higher expectations across several usefulness-related values. They notably emphasized health as a critical factor (older adults: mean 4.02, SD 0.79 vs younger adults: mean 3.60, SD 1.03; t_411_=–4.64; *P*<.001; *d*=0.46), highlighting their preference for health-related features. Additionally, novelty was rated significantly higher by older adults (mean 3.97, SD 0.74) than by younger adults (mean 3.45, SD 0.89; t_411_=–6.44; *P*<.001; *d*=0.62), suggesting an attraction to fresh and innovative experiences.

In terms of emotional and social benefits, older adults consistently placed greater importance on convenience (mean 3.91, SD 0.62), fun (mean 3.85, SD 0.70), and friendship (mean 3.37, SD 0.72) compared to younger adults (convenience: mean 3.59, SD 0.95; fun: mean 3.46, SD 0.96; and friendship: mean 3.03, SD 0.97). They also rated confidence (mean 3.66, SD 0.81) and a sense of belonging (mean 3.54, SD 0.79) significantly higher than younger adults (confidence: mean 3.21, SD 0.91 and belonging: mean 3.25, SD 1.01). While not statistically significant, both age groups similarly recognized the emotional benefits related to memories, though these were less differentiated between them.

##### Aesthetic and Gamification Components

In evaluating aesthetic and gamification components, older adults generally expressed a greater interest in several features compared to younger adults. Specifically, older adults showed a significantly stronger interest in customization options like avatars and personalized profiles (older adults: mean 3.28, SD 0.89 vs younger adults: mean 2.92, SD 1.08; t_411_=–3.69; *P*<.001; *d*=0.36), suggesting a desire for more personalized UXs.

A notable difference also emerged in friend integration, with older adults showing a much higher preference for linking the app with external platforms like KakaoTalk or Facebook (mean 3.64, SD 0.88 vs mean 3.04, SD 1.17; t_411_=–5.87; *P*<.001; *d*=0.57). This highlights the importance of seamless social connectivity for older users. Furthermore, older adults reported greater interest in immersive meeting spaces (eg, augmented reality or virtual reality features) compared to younger adults (mean 3.12, SD 0.87 vs mean 2.91, SD 1.12; t_411_=–2.12; *P*=.03; *d*=0.20), suggesting that immersive environments may enhance their digital engagement.

While older adults appreciated rewards like points and badges more than younger adults (mean 3.49, SD 0.84 vs mean 3.32, SD 1.12), this difference was not statistically significant (t_411_=–1.74; *P*=.08; *d*=0.17). Finally, visual appeal (eg, icons and backgrounds) was rated similarly across both age groups (older adults: mean 3.50, SD 0.81 and younger adults: mean 3.46, SD 0.93; t_411_=–0.47; *P*=.64; *d*=0.04), indicating a shared preference for aesthetically pleasing user interface (UI).

Table 1 summarizes generational differences in attitudes toward intergenerational communication, perceptions of app necessity and willingness to use, motivational factors, and preferred app features.

**Table 1:** Comparison of younger and older adults’ attitudes, perceptions, motivations, and preferred features for an intergenerational communication app (N=413).

					Younger (n=202), mean (SD)			Older (n=211), mean (SD)			*t* test (*df*=411)			*P* value
**Attitudes toward intergenerational communication**														
	Interest in cross-generational communication			3.61 (0.81)			*3.87 (0.63)* ^a^			–*3.63*			*<.001*	
	Perceived importance of cross-generational communication			3.86 (0.82)			*4.05 (0.66)*			–*2.59*			*.01*	
**Perceived necessity and willingness to use an intergenerational communication app**														
	Perceived necessity of an intergenerational communication app			3.47 (0.83)			*3.82 (0.73)*			–*4.54*			*<.001*	
	Willingness to use the app			3.17 (0.87)			*3.81 (0.71)*			–*8.17*			*<.001*	
**Motivational factors for app use**														
	Social communication			3.61 (0.81)			*3.87 (0.63)*			–*3.63*			*<.001*	
	Passing time (relieving boredom)			3.19 (1.01)			*2.97 (0.86)*			*2.38*			*.01*	
	Entertainment and enjoyment			3.19 (1.03)			*3.50 (0.73)*			–*3.52*			*<.001*	
	Exchanging necessary information or opinions			3.86 (0.82)			*4.05 (0.66)*			–*2.59*			*.01*	
	Reducing loneliness			2.89 (1.12)			*3.10 (0.89)*			–*2.10*			*.04*	
	Health management			3.31 (1.02)			*3.77 (0.88)*			–*4.90*			*<.001*	
	Discussing concerns not easily shared with same-generation peers			3.59 (0.94)			*3.06 (0.94)*			*5.73*			*<.001*	
	Cultural exchange			3.44 (0.94)			*3.72 (0.72)*			–*3.39*			*.001*	
**Preferred app features**														
	**Perceived ease of use**													
		Ease of access to information or content	4.00 (0.73)			*4.14 (0.60)*			–*2.12*			*.03*		
		Immediate accessibility	3.84 (0.85)			3.86 (0.70)			–0.26			.79		
		Reliability of provided information	4.26 (0.86)			*4.41 (0.66)*			–*1.98*			*.048*		
		Clarity and accuracy of information	4.16 (0.83)			4.26 (0.68)			–1.34			.18		
		Relevance and appropriateness of content	4.05 (0.75)			4.12 (0.69)			–0.99			.32		
		Simplicity and readability of content	3.91 (0.93)			4.03 (0.78)			–1.42			.16		
		Engagement and entertainment value	3.87 (0.79)			3.81 (0.75)			0.79			.43		
	**Perceived usefulness**													
		Convenience	3.59 (0.95)			*3.91 (0.62)*			–*4.03*			*<.001*		
		Fun	3.46 (0.96)			*3.85 (0.70)*			–*4.70*			*<.001*		
		Friendship	3.03 (0.97)			*3.37 (0.72)*			–*4.03*			*<.001*		
		Calmness	3.44 (1.01)			3.58 (0.74)			–1.60			.11		
		Confidence	3.21 (0.91)			*3.66 (0.81)*			–*5.30*			*<.001*		
		Sense of belonging	3.25 (1.01)			*3.54 (0.79)*			–*3.24*			*.001*		
		Memories	3.48 (1.01)			3.46 (0.86)			0.22			.83		
		Satisfaction	3.43 (0.95)			*3.63 (0.76)*			–*2.36*			*.019*		
		Health	3.60 (1.03)			*4.02 (0.79)*			–*4.64*			*<.001*		
		Novelty	3.45 (0.89)			*3.97 (0.74)*			–*6.44*			*<.001*		
**Aesthetics and gamification components**														
	Rewards (points, badges, levels)			3.32 (1.12)			3.49 (0.84)			–1.74			.08	
	Customization (avatars, profiles)			2.92 (1.08)			*3.28 (0.89)*			–*3.69*			*<.001*	
	Friend integration (KakaoTalk, Facebook)			3.04 (1.17)			*3.64 (0.88)*			–*5.87*			*<.001*	
	Immersive meeting spaces (AR^b^ or VR^c^)			2.91 (1.12)			*3.12 (0.87)*			–*2.12*			*.03*	
	Visual appeal (icons, backgrounds)			3.46 (0.93)			3.5 (0.81)			–0.47			.64	

^a^Values in italics format indicate statistically significant differences at *P*<.05.

^b^AR: augmented reality.

^c^VR: virtual reality.

#### Qualitative Responses on Intergenerational Information and Conversation Topics

Participants expressed strong interest in sharing hobbies, health tips, financial advice, travel experiences, and pet care—topics that may serve as effective entry points for intergenerational dialogue. These findings support the use of thematically structured discussion boards to guide user-generated content, providing a flexible yet focused environment for sustained and reciprocal interaction based on shared interests.

### Phase 2: Define; UX Priorities and Design Implications

This phase translated the findings from phase 1 into concrete design priorities for the CoGen prototype. Quantitative and qualitative analyses revealed distinct generational differences in usability preferences, interaction styles, and motivational drivers.

#### Generational Differences in UX Preferences

Older adults emphasized clarity, structure, and accessibility. For instance, 73.4% (n=155) of older users selected “easy navigation” as a top priority compared to 52.6% (n=106) of younger users. They also rated the need for structured information layouts as significantly higher (mean 4.26, SD 0.62) than younger adults (mean 3.68, SD 0.81; t_411_=–4.57; *P*<.001).

In contrast, younger adults favored engaging and dynamic elements. A total of 68.3% (n=138) of younger users prioritized “fun and dynamic design” compared to 41.2% (n=87) of older adults. They also showed greater interest in gamified features such as conversation streaks and badges (mean 3.92, SD 0.77) than older users (mean 3.41, SD 0.84; t_411_=–3.21; *P*=.002).

Both groups expressed strong interest in structured discussion themes, though topic preferences varied. Older adults preferred themes such as health, pets, and daily life tips, while younger users leaned toward travel, hobbies, and financial advice. Open-ended responses supported these findings, with one older adult stating, “If I could talk about gardening or health tips, I would join more often,” and a younger participant noting, “It’s fun when it feels like social media, not a lecture.”

#### Design Implications for the CoGen App

These generational patterns directly informed the prototype development of CoGen. The interface was designed to balance simplicity with visual appeal, offering a minimalist layout suited to older users while providing customizable elements to satisfy younger users’ preferences. Accessibility features such as optional auditory cues (eg, notification sounds and voice-based guidance) were included to accommodate diverse needs without overwhelming the user. Thematic discussion boards organized around popular topics such as health, hobbies, and finance were implemented to support guided yet flexible interactions. Additionally, participatory components like conversation streaks, shared milestones, and feedback prompts were incorporated to foster social engagement and maintain user interest over time, especially among younger participants.

### Phase 3: Develop; Finalized Features and User-Driven Adjustments

This phase focused on translating insights from earlier phases into a functional CoGen prototype through iterative development. Drawing on findings from phases 1 and 2, the design process integrated key generational preferences into the app’s structure and functionality, followed by small-scale usability testing and refinement.

#### UI or UX Design and Wireframing Outcomes

Based on generational needs identified in earlier phases, the CoGen prototype was wireframed with a focus on accessibility for older adults and visual engagement for younger users. Older participants responded positively to features such as enlarged font sizes and high-contrast modes, frequently describing them as “easier to read” and “less tiring on the eyes.” In contrast, younger users favored vibrant color schemes and modern layouts, stating that the design “felt more like a social space.” The home screen—structured around 5 core functions—was perceived as intuitive and manageable across age groups. Additionally, the inclusion of a numeric level indicator with a horizontal progress bar was initially well received as a simple motivator, though later feedback suggested a need for more emotionally resonant visualizations.

#### Prototype Development and Feature Integration Outcomes

##### Prototype Features and User Feedback

The functional prototype incorporated features designed to foster sustained intergenerational engagement, informed by user needs identified in earlier phases. Feedback from older and younger participants highlighted differing priorities—older adults emphasized meaningful conversation and ease of use, while younger users favored interactivity and entertainment-driven engagement. Four key features were evaluated and refined based on user reactions.

##### Discussion Boards

Theme-based forums were designed around topics such as hobbies, travel, and health. This structure aimed to support older users’ desire for organized, topic-specific discussions while offering younger users spaces for more casual and spontaneous interaction.

##### Conversation Triggers

Participatory elements, including prompts and quizzes, were introduced to ease the initiation of conversations. These features were intended to reduce entry barriers and encourage mutual storytelling, particularly among younger users who prefer playful engagement.

##### 1:1 Friend Matching

A proprietary friend-matching feature was included to support more personalized interactions, especially for older adults who seek deeper, one-on-one conversations. This function was designed as an in-app alternative to social media integration, minimizing complexity and maintaining user privacy.

##### Generational Identity Icons

**Figure 2:**
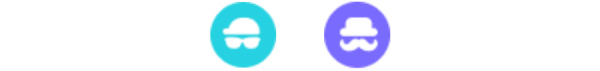
Generational identity icons: youth (left) and older adult (right).

To promote age-aware interaction, generation-specific icons (eg, youth and older adult symbols) were added next to each user ID. This visual cue enabled users to identify the generational background of their conversation partners, fostering mutual understanding and respectful communication across age groups (Figure 2).

##### Progression-Based Gamification

To encourage consistent app engagement, a light-touch gamification system was implemented. Users could earn points through participation (eg, posting or commenting), which contributed to levels and badges. The system was intentionally noncompetitive, reflecting older users’ preference for collaborative over competitive dynamics.

#### Preliminary Usability Testing and Prototype Refinement

##### Preliminary Testing Outcomes

Initial usability testing with 8 participants (5 younger adults and 3 older adults) revealed critical insights that informed refinements to the CoGen prototype. Participants evaluated the app’s overall appeal, navigability, and clarity of features. Several features were refined based on this feedback.

##### Branding and Identity

The app was named “CoGen” to reflect its focus on cogenerational engagement. The elephant logo—symbolizing wisdom, memory, and social bonds—resonated with participants’ desire for meaningful and emotionally grounded experiences.

##### Weekly Question Feature

Participants favored prompts that evoked shared memories and generational perspectives. Based on this input, the “Weekly Question” was redesigned to include open-ended questions that invite storytelling and nostalgic reflection.

##### Engagement Indicator

The original numeric level and progress bar were perceived as impersonal. In response, the engagement indicator was redesigned into a metaphorical growth journey—a seed blossoming into a flower—offering a more intuitive and emotionally resonant representation of user progress, especially appreciated by older participants.

##### Tone of Feature Labels

**Figure 3:**
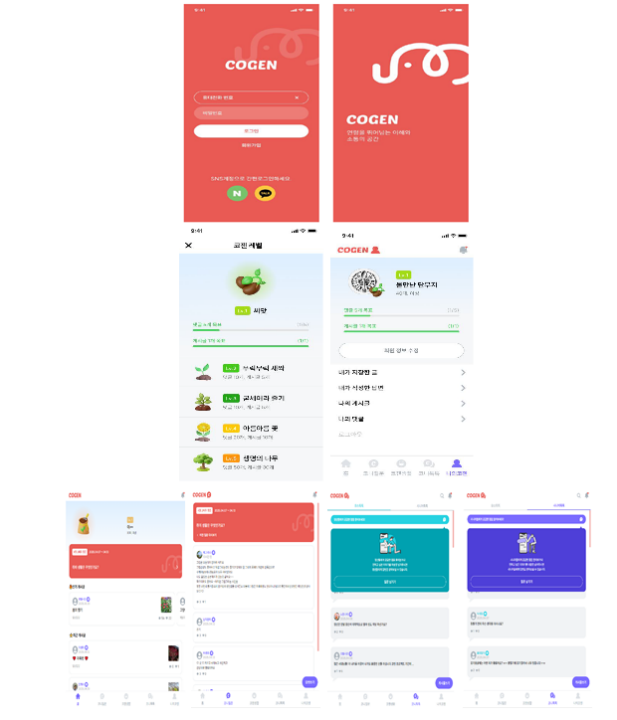
Final version of the CoGen app interface, displaying (from top to bottom): the login screen, level indicator, home screen with “Coni’s Question,” and “Cogen TalkTalk” features.

Younger participants emphasized the importance of a conversational tone. In response, feature names were modified to be more inviting and playful. For example, the discussion board was renamed “CoGen Life,” and the weekly prompt became “Coni’s Question.” These refinements finalized the CoGen prototype by incorporating user-driven feedback into its core features and design, setting the stage for real-world testing in the deliver phase (Figure 3).

### Phase 4: Deliver; Real-World Testing Outcomes and Final Revisions

#### User Engagement Patterns Based on Activity Log Data

To assess the feasibility and user engagement of the CoGen platform during the 2-week intervention, activity log data were analyzed. Participants logged into the app an average of 31.43 (SD 10.56) times, with a total of 2975 posts and comments generated—equivalent to an average of 76.28 (SD 20.13) entries per user. This indicates both frequent access and active participation in intergenerational discussions.

Engagement with in-app features—such as quizzes and feedback prompts—was also high, with 80.3% (n=31) of users completing them at least once. The dropout rate remained low at 5.3% (n=2), and activity levels were stable throughout the period, suggesting sustained engagement.

#### UX: Functional Satisfaction

Participants reported moderate to high levels of comfort in cross-generational conversations (mean 3.62, SD 0.81) and relatively high overall satisfaction (mean 3.82, SD 0.76) with intergenerational conversations via the app.

Regarding emotional engagement, users reported high levels of enjoyment (mean 3.74, SD 0.85) and happiness (mean 3.79, SD 0.77), with perceived interest being the highest measured variable (mean 4.05, SD 0.79). While the perceived sense of similarity or affinity was moderate (mean 3.46, SD 0.94), it still indicated a positive trend in user closeness.

Conversely, ease of access received a comparatively lower rating (mean 2.59, SD 0.88), indicating potential navigation difficulties. Similarly, perceived understanding of content was moderate (mean 3.23, SD 0.71), suggesting opportunities for optimizing content presentation and guidance.

#### Impact on Perceived Necessity, Use Intentions, and Recommendation Willingness

No statistically significant changes were observed in participants’ perceived necessity (t_37_=1.58; *P*=.13; *d*=0.45) or intention to continue using the app (t_37_=0.81; *P*=.43; *d*=0.25; Table 2). However, perceived necessity was already high before the intervention (mean 3.70, SD 0.73; mean 4.05, SD 0.83), suggesting a potential ceiling effect. Intention to use remained moderate but stable (mean 3.30, SD 0.86; mean 3.50, SD 0.69). Participants also showed a moderate willingness to recommend the app (mean 3.55, SD 1.01), reflecting its perceived value in fostering cross-generational communication.

**Table 2:** Changes in key variables before and after the intervention (n=39).

Variable	Pre, mean (SD)	Post, mean (SD)	*t* test (*df*=37)	*P* value
Intergenerational interaction	2.85 (1.04)	3.85 (0.75)	–3.82	.001
Intergenerational understanding	1.42 (0.40)	3.02 (0.72)	–10.26	<.001
Perceived social support	3.03 (0.53)	3.85 (0.78)	–5.53	<.001
Depressive symptoms	1.85 (0.36)	1.82 (0.37)	0.4	.69
Perceived necessity of the app	3.70 (0.73)	4.05 (0.83)	1.58	.13
Intention to continue using the app	3.30 (0.86)	3.50 (0.69)	0.81	.43
Willingness to recommend the app (post)	—^a^	3.55 (1.01)	—	—

^a^Not available.

#### Changes in Intergenerational Communication: Interaction and Understanding

Paired-sample 2-tailed *t* tests revealed statistically significant improvements in both intergenerational interaction and understanding after the 2-week intervention (Table 2). Intergenerational interaction increased significantly (t_37_=–3.82; *P*=.001; *d*=0.52), signifying more frequent and natural conversations. Intergenerational understanding also showed a substantial and statistically significant improvement (t_37_=–10.26; *P*<.001; *d*=1.16), indicating that the app effectively enhanced empathy toward different generational perspectives.

#### Impact on Psychosocial Outcomes: Social Support and Depressive Symptoms

Perceived social support significantly increased following 2 weeks of CoGen app use (t_37_=–5.53; *P*<.001; *d*=0.66), suggesting that the app strengthened emotional bonds (Table 2). In contrast, depressive symptoms showed no statistically significant change (t_37_=0.40; *P*=.69; *d*=–0.06), implying that while social connectedness improved, immediate effects on emotional well-being were not observed within the short time frame.

#### Qualitative Insights From the FGI

To supplement the survey findings, an FGI was conducted, revealing that participants from both age groups found the app emotionally enriching and socially meaningful. They especially appreciated the structured yet open environment that supported respectful and thoughtful dialogue.

##### Mutual Adaptation and Respectful Communication

Participants reported that their attitudes toward intergenerational communication shifted positively during the 2-week period. Younger adults initially expressed concern about potential generational conflict but noted that the platform encouraged the use of respectful and considerate language. In turn, older adults also exercised caution to avoid appearing condescending or outdated, demonstrating a shared effort to communicate with mutual respect. This digital form of etiquette was recognized by both groups as an important cultural adjustment, especially when engaging with unfamiliar age cohorts.

##### Psychosocial Gains and Shifting Perceptions

The app served as a space where younger users could seek emotional support, practical advice, and broader perspectives from older participants. Many reported gaining comfort, validation, and a renewed appreciation for the insights of older generations. Meanwhile, older adults described feeling revitalized by the interactions, often expressing surprise at the empathy and openness of younger users. They also viewed the app as a rare opportunity to reflect on their own life stories while learning about new cultural trends and digital practices.

##### Common Recognition of Generational Diversity

Participants across age groups highlighted how the experience disrupted stereotypical perceptions. Younger users came to recognize the diversity among older adults, moving beyond generalized assumptions. Conversely, older participants reported gaining a more nuanced understanding of younger people’s struggles and viewpoints, fostering empathy and solidarity.

##### Conditions for Sustained Use

Despite the positive feedback, participants noted several functional improvements necessary for long-term engagement. Many called for real-time chat features, simplified login processes, and improved content navigation to support more spontaneous and efficient communication. Additionally, participants expressed that future iterations of the app should focus on enabling users to “gain something meaningful” through dialogue—such as wisdom, emotional encouragement, or practical advice. Both groups emphasized the need for structured prompts and shared interests (eg, health and hobbies) to sustain motivation.

##### Design Suggestions

Users also offered specific UI or UX recommendations, including expressive elements like emojis or stickers, and personalized notification systems. These enhancements were seen as important for maintaining emotional resonance and playful interaction across generations, supporting more engaging and expressive communication for all users.

## Discussion

### Principal Findings

This study provides preliminary evidence that the CoGen app may contribute to improvements in interaction, perceived social support, and comfort in cross-generational conversations. These results align with previous findings, indicating that intentional and structured opportunities for age-diverse contact can reduce prejudice and strengthen social bonds [5,7]. Specifically, the observed improvements in dialogue across generational lines are consistent with the principles of the contact hypothesis, suggesting that digital platforms hold potential spaces for meaningful engagement when designed to facilitate regular and constructive exchanges among different age groups.

Improvements in perceived social support align with previous research, indicating that older adults benefit from increased digital communication by maintaining social ties and mitigating feelings of isolation [36,37]. Younger adults also reported gaining fresh perspectives and insights from older generations, suggesting that positive, bidirectional exchanges can foster empathy and reduce generational stereotyping. This finding is aligned with prior studies, highlighting the significance of qualitatively rich interactions over mere contact frequency in achieving prejudice reduction and mutual understanding [8].

Furthermore, the study’s results suggest that participants experienced an increased sense of comfort and satisfaction in conversations with individuals from different generations. These findings tentatively support the view that intergenerational communication can be nurtured beyond physical copresence, especially as digital literacy expands among older adults. Although ease of access scored relatively lower, it underlines the importance of continued efforts to design age-friendly digital interfaces that reduce navigation barriers and enhance older adults’ confidence in adopting new technologies [38]. Participants specifically emphasized the need for auto-login features, clearer content categorization, and easier navigation to locate new or popular posts. Older adults in particular faced challenges in understanding the interface layout and expressed a desire for simplified, intuitive pathways to access discussions. To enhance emotional engagement, suggestions included incorporating expressive elements such as emojis and stickers.

A key emergent finding from the FGIs was the positive reception of the generational badge feature, which visually distinguished older and younger users. Despite initial concerns about reinforcing age-based divisions, many participants noted that it surprisingly encouraged respectful and age-sensitive communication, even in anonymous interactions. Younger users, in particular, reported feeling a greater sense of obligation to maintain courtesy, while older users appreciated being addressed with politeness. These findings suggest that CoGen’s features organically aligned with Korean cultural norms rooted in Confucian values, such as intergenerational respect and age-based etiquette. Rather than merely enabling conversations, the app fostered a communicative atmosphere marked by mutual consideration—an emergent property shaped by both interface design and cultural expectations.

Moreover, younger participants shared in the FGIs that the app served as a psychologically safe space to seek advice or express concerns they might hesitate to bring up in face-to-face settings (eg, with parents). In contrast to offline environments where social expectations and *noonchi*—the culturally embedded sensitivity to others’ feelings—can act as barriers, and the app allowed for open and honest exchanges without fear of breaching generational decorum. This illustrates how CoGen’s design facilitated not just communication, but culturally meaningful connection, by providing users with both symbolic cues for respectful interaction and a space that eased the emotional burden of navigating age hierarchies.

In contrast to many existing digital interventions that often emphasize structured prompts or 1-way content delivery, CoGen was specifically designed to support user-generated content and dynamic, reciprocal communication between generations. Most prior platforms still lack robust mechanisms for sustained, bidirectional interaction, and despite increasing digital literacy among older adults [13,14], many fall short in addressing the nuanced interaction needs and co-design preferences of both younger and older users. Addressing this critical gap, CoGen positions itself as a hybrid model bridging formal programs and informal social interaction—an area still underdeveloped in the digital aging landscape. The app integrates both synchronous and asynchronous communication, encourages user-generated content, and offers structured prompts to support long-term engagement. This approach is strongly reinforced by CoGen’s adherence to principles of inclusive co-design and iterative user-centered development [15]. As literature increasingly highlights the digital literacy of older adults [39] and the importance of inclusive co-design [40], CoGen specifically reflects these principles through its iterative development and participatory UX testing. These practices not only improve usability but also enhance users’ sense of ownership and emotional attachment to the platform, further supporting long-term adoption.

Despite its promising outcomes, the relatively lower usability scores underscore the need for improved accessibility. Participants emphasized visual clarity, reduced click-depth, and features like auto-login and richer emotional expression tools. These findings reinforce the importance of UCD and inclusive codevelopment, ensuring that intergenerational digital tools remain not only functional but also emotionally engaging and culturally responsive. CoGen’s iterative design process and hybrid communication structure offer a promising model—particularly in societies like Korea, where age-based etiquette shapes interaction norms—by fostering respectful and psychologically safe exchanges across generations.

### Theoretical and Practical Implications

The structured features of CoGen—such as discussion boards, quizzes, and shared achievements—appeared to reduce social distance and foster mutual understanding across generations. These affordances reflect core principles of empathy-building and cooperative engagement found in offline intergenerational programs [7,41], while offering scalable digital alternatives. Although the contact hypothesis was not directly tested, several of its key conditions—such as equal status, shared goals, and structured interaction—were naturally embedded in the design of CoGen. These elements may have contributed to the observed improvements in mutual understanding and reduction of age-based bias. This highlights the value of grounding digital intervention development not only in UCD principles but also in theoretically informed frameworks, which together can enhance both usability and psychosocial outcomes.

As digital connectivity expands globally, platforms like CoGen hold promise for supporting intergenerational relationships, despite physical separation. This function aligns with models of digital age-friendly communities [38] and aging-in-place strategies [42], both of which emphasize the role of sustained social engagement in promoting healthy aging. By enabling remote yet meaningful interactions, CoGen represents a digital extension of the World Health Organization’s vision of inclusive, communicative, and socially connected environments for older adults [43].

The observed improvement in perceived social support reinforces previous findings that emotionally resonant communication enhances older adults’ well-being and cognitive engagement [44,45]. CoGen’s focus on sustained dialogue may thus contribute to improved life satisfaction and emotional resilience. In line with World Health Organization guidelines [42], promoting regular digital interactions through accessible platforms can also support independent living and active aging.

In the Korean context, where Confucian values such as filial piety and age-based respect remain culturally salient, CoGen’s emphasis on structured, respectful dialogue aligns well with traditional norms of intergenerational harmony. Participant feedback indicated that the platform created a safe space for expressing concerns—especially for younger users—while maintaining cultural expectations around age hierarchy. This suggests the culturally adaptive potential of digital tools to bridge generational gaps by balancing modern communication and traditional values.

Finally, design feedback points to future opportunities for enhancing cross-generational engagement. Participants emphasized the value of algorithm-based matching systems to facilitate interest-based dialogue, as well as the need for simplified UI or UX for older adults. Incorporating expressive features such as emojis and stickers may further support engagement among younger users. These insights highlight the importance of participatory, multigenerational design frameworks in creating emotionally inclusive and functionally accessible digital environments.

### Limitations and Future Research

Despite its promising findings, this study has several limitations that offer clear directions for future research. First, the sample size was relatively small, and the 2-week intervention period was short. These factors may limit the generalizability of our findings and the understanding of long-term engagement. Future research should involve large-scale, longitudinal studies to track sustained engagement and relational outcomes over an extended period. Such studies would allow us to verify if the observed benefits persist and evolve across more diverse cultural and demographic contexts.

Second, our design could be enhanced by incorporating real-time interaction and deeper personalization. While the app effectively supported asynchronous communication, participants expressed a strong desire for synchronous features like direct messaging. Moreover, to move beyond basic usability and better capture the emotionally sensitive nature of intergenerational dialogue, future design cycles will integrate formal tools from design thinking. Specifically, we plan to use empathy maps and user journey visualizations to inform the development of more sophisticated features, such as adaptive dialogue prompts and sentiment-aware interaction flows, thereby creating a more responsive and emotionally resonant UX.

Third, our reliance on self-reported measures could be complemented by objective data. While valuable, self-reports on comfort or connection may not capture the full picture of user behavior. Future studies could create a more holistic understanding by integrating use analytics—such as the frequency, duration, and content analysis of interactions—with self-report data to triangulate findings on how digital communication truly evolves.

Finally, several key constructs were measured using single-item indicators. This was a deliberate methodological choice grounded in both practical and theoretical considerations. Rather than combining conceptually distinct items into composite scores, we retained item-level granularity to capture feature-specific responses that could directly inform app development. This approach aligns with prior research indicating that single-item measures can be valid and useful, especially when constructs are concrete, unidimensional, and behaviorally specific [28]. Although the items were carefully adapted from previously validated multi-item instruments, we acknowledge that multi-item scales generally offer higher reliability. Future studies should incorporate established multi-item measures to validate the findings and enable more robust analyses, such as latent variable modeling, to advance theoretical understanding.

By addressing these limitations, future research can build upon our work to design inclusive digital interventions that foster meaningful connections and contribute to the broader goals of healthy aging and creating age-friendly societies.

## Abbreviations

FGI: focus group interview
UCD: user-centered design
UI: user interface
UX: user experience
